# Methamphetamine induces Shati/Nat8L expression in the mouse nucleus accumbens via CREB- and dopamine D1 receptor-dependent mechanism

**DOI:** 10.1371/journal.pone.0174196

**Published:** 2017-03-20

**Authors:** Kyosuke Uno, Toh Miyazaki, Kengo Sodeyama, Yoshiaki Miyamoto, Atsumi Nitta

**Affiliations:** Department of Pharmaceutical Therapy and Neuropharmacology, Faculty of Pharmaceutical Sciences, Graduate School of Medicine and Pharmaceutical Sciences, University of Toyama, Toyama, Japan; University of South Carolina School of Medicine, UNITED STATES

## Abstract

*Shati/Nat8L* significantly increased in the nucleus accumbens (NAc) of mice after repeated methamphetamine (METH) treatment. We reported that *Shati/Nat8L* overexpression in mouse NAc attenuated METH-induced hyperlocomotion, locomotor sensitization, and conditioned place preference. We recently found that *Shati/Nat8L* overexpression in NAc regulates the dopaminergic neuronal system via the activation of group II mGluRs by elevated *N*-acetylaspartylglutamate following *N-*acetylaspartate increase due to the overexpression. These findings suggest that *Shati/Nat8L* suppresses METH-induced responses. However, the mechanism by which METH increases the *Shati/Nat8L* mRNA expression in NAc is unclear. To investigate the regulatory mechanism of *Shati/Nat8L* mRNA expression, we performed a mouse *Shati/Nat8L* luciferase assay using PC12 cells. Next, we investigated the response of METH to *Shati/Nat8L* expression and CREB activity using mouse brain slices of NAc, METH administration to mice, and western blotting for CREB activity of specific dopamine receptor signals in vivo and ex vivo. We found that METH activates CREB binding to the *Shati/Nat8L* promoter to induce the *Shati/Nat8L* mRNA expression. Furthermore, the dopamine D1 receptor antagonist SCH23390, but not the dopamine D2 receptor antagonist sulpiride, inhibited the upregulation of *Shati/Nat8L* and CREB activities in the mouse NAc slices. Thus, the administration of the dopamine D1 receptor agonist SKF38393 increased the *Shati/Nat8L* mRNA expression in mouse NAc. These results showed that the *Shati/Nat8L* mRNA was increased by METH-induced CREB pathway via dopamine D1 receptor signaling in mouse NAc. These findings may contribute to development of a clinical tool for METH addiction.

## Introduction

Addiction and abuse of drugs such as methamphetamine (METH) are social problems worldwide [1]. It is well-known that METH induces specific behavioral responses such as hyperlocomotion, locomotor sensitization, and conditioned place preference in mice [2] and increases dopamine levels in the synaptic clefts [3, 4]. Many psychostimulative properties induced by METH increase dopamine levels via the inhibition of reuptake through the dopamine transporter on presynapses in specific regions, including the nucleus accumbens (NAc) [5]. Projection of the dopaminergic neuronal system from the ventral tegmental area (VTA) to NAc is associated with METH dependence [6]. Medium spiny neurons that express dopamine D1 or D2 receptors (D1 or D2-MSN) in NAc are GABAergic neurons, and D1-MSN of NAc projected to VTA and strongly preferred the GABA neurons of VTA [7]. These reports demonstrated that projection from VTA to NAc is critical for METH addiction and that D1-MSN at NAc may affect the activity of VTA dopaminergic neurons.

We previously showed that Shati, which is a novel molecule containing a conserved sequence of the *N-*acetyltransferase superfamily, was elevated in NAc of mice administered METH [8]. Ariyannur and coworkers reported that Shati generated *N-*acetylaspartate (NAA) from aspartate and acetyl-CoA as *N*-acetyltransferase 8-like protein (Nat8l) [9]. Next, we renamed the molecule from Shati to Shati/Nat8L [10–12]. NAA passes through conversion to *N*-acetylaspartylglutamate (NAAG) as the agonist of metabotropic glutamate receptor type 3 (mGluR3) [13].

Knockdown of *Shati/Nat8L* by the treatment of mice with antisense oligonucleotides showed that METH-induced behavioral alterations were enhanced and dopamine levels in NAc were increased [8]. We recently reported that the overexpression of *Shati/Nat8L* by adeno-associated virus vector in NAc attenuated METH-induced responses by controlling the dopaminergic system via the activation of mGluR3 [11]. Furthermore, *Shati/Nat8L* promoted the localization of dopamine D1 receptor to the cell surface via association with the adaptor protein-2 complex [14]. Thus, the expression of *Shati/Nat8L* contributes to behaviors depending on dopaminergic neurons and molecular localizations with dopamine receptors. These findings indicate that the regulatory system of the expression of *Shati/Nat8L* in NAc could be critical for dopamine-induced dependent behaviors. However, the detailed mechanism of the expression of *Shati/Nat8L* in the mouse brain is unclear.

In this study, we investigated METH-induced increases in *Shati/Nat8L* mRNA to understand the expression mechanism and identified the Shati/Nat8L promoter of the mouse gene. We found that the transcriptional factors cAMP response element-binding protein (CREB) and nuclear factor kappa-light-chain-enhancer of activated B cells (NF-κB) may bind to the promoter region of *Shati/Nat8L* and that the induction of the *Shati/Nat8L* mRNA expression is regulated by CREB via the activation of dopamine D1 receptors; furthermore, we discussed about how a clarification of the regulatory mechanism of *Shati/Nat8L* may contribute to the development of a clinical tool for METH addiction.

## Materials and methods

### Drugs and reagents

METH hydrochloride was purchased from Dainippon Sumitomo Pharmaceutical Co. (Osaka, Japan). SKF38393 hydrobromide, quinpirole hydrochloride, and sulpiride were purchased from Tocris Bioscience (Bristol, UK). SCH23390 hydrochloride was acquired from Sigma-Aldrich (St. Louis, MO). The doses of these drugs were the same as those used in previous studies SKF38393 (0.5 mg/kg) [14], quinpirole hydrochloride (0.05 mg/kg, 0.5 mg/kg), SCH23390 hydrochloride (0.5 mg/kg) [15, 16], and sulpiride (20 mg/kg) [17].

### Animals

Male C57BL/6J inbred mice were acquired from Nihon SLC Inc. Japan (Shizuoka, Japan) were 8 weeks old. Mice and kept in the animal institute of University of Toyama were in a temperature- and humidity-controlled environment under a 12-h light/12-h dark cycle (lights on at 8:00) and had ad libitum access to food and water. The health and welfare of the animals was monitored by staff at least once a day. All mice were quickly decapitated by animal guillotine without feeling any suffering, since the fresh brain tissues were needed for the isolation of mRNA or brain slices. This procedure were done without anesthesia to avoid the effect of anesthetic drugs. All procedures followed the National Institute of Health Guideline for the Care and Use of Laboratory Animals (NIH publication No. 85–23, revised in 1996) and were approved by the committee for Animal Experiments of the University of Toyama (Permit Number A2015-PHA23). In the permission, It has been stated that even during the experiment period, if an animal experiences symptoms of torture (self-injury behavior, abnormal posture, crying etc.) and rapid weight loss (more than 20% in several days), take measures of euthanasia, with sodium pentobarbital (120mg/kg). However no mice were observed in such a situation in this study.

### Cell culture

Rat PC12 cells were obtained from RIKEN (Ibaraki, Japan) and used within 12 passages of the original vial. PC12 cells were grown in high–glucose Dulbecco’s eagle medium (D-MEM) (Wako Pure Chemicals, Osaka, Japan) supplemented with 10% fetal bovine serum (FBS) (Nichirei Biosciences, Tokyo, Japan), 5% horse serum (Gibco BRL, Palo Alto, CA), and 1% penicillin/streptomycin (PS). Mouse neuroblastoma-cells (Neuro2a) were obtained from DS Pharma Biomedical (Osaka, Japan) and within 12 passages of the original vial. Neuro2a cells were grown in D-MEM (low glucose) (Wako Pure Chemicals) supplemented with 10% FBS and 1% PS. Cell cultures were all maintained at 37°C in a humidified atmosphere containing 5% CO2.

### Quantitative RT-PCR

Quantitative RT-PCR for *Shati/Nat8L* was performed according to a method previously reported [14]. In brief, total RNA extraction was performed using TRIsure (Meridian Life Science Company, Memphis, TN). Total RNA extracted from NAc tissue of mice, PC12 cells, and Neuro2a were transcribed into cDNA using the Prime Script RT reagent kit (Takara, Shiga, Japan) according to the manufacturer’s instructions. The reaction was performed at 37°C for 20 min in a total volume of 10 μl and inactivated at 85°C for 10 s. Real-time PCR was performed using SYBR-Green-based reagents (Thunder Bird Sybr qPCR Mix, Toyobo, Tokyo, Japan) and a Takara Dice Real Time System (Takara). The reaction was performed according to the cycling protocol (5 min heat activation of the enzyme at 95°C, 40 cycles of denaturation at 95°C for 20 s, annealing at 60°C for 20 s, and extension at 72°C for 20 s). The following primer sequences were used for PCR: 5′-GTGATTCTGGCCTACCTGGA-3′ (forward) and 5′-CCACTGTGTTGTCCTCCTCA-3′ (reverse) as mice *Shati/Nat8L* primers for mice brain tissue and Neuro2a, 5′-GTGATTCTGGCCTACCTGGA-3′ (forward) and 5′-CCACTGTGTTGTCTTCCTCA-3′ (reverse) as Rattus *Shati/Nat8L* primers for PC12 cells, 5′-ACCCTGAAGTGCTCGACATC-3′ (forward,) and 5′-AGGAAGGCCTTGACCTTTTC-3′ (reverse) as mice 36B4 primers and 5′-CTCAGTGCCTCACTCCATCA-3′ (forward) and 5′-CTTCCTTTGCTTCGACCTTG-3′ (reverse) as Rattus 36B4 primers for PC12 cells.

### Design and production of vectors

Production of *Shati/Nat8L* luciferase vector was performed according to a method previously reported [18]. To perform the luciferase assay, we produced *Shati/Nat8L* promoter-driven luciferase vectors from the pGL3-Basic Vector (Promega, Madison, WI) after in silico analysis on TF search: Searching Transcription Factor-Binding Sites (ver 1.3: http://diyhpl.us/~bryan/irc/protocol-online/protocol-cache/TFSEARCH.html). Because we found putative binding sites of major transcriptional factors, specifically, AP-1, NF-κB, and CREB, by in silico analysis of TF search in the *Shati/Nat8L* promoter, we produced various expression vectors that subsequently had their *Shati/Nat8L* promoter region deleted. *Shati/Nat8L* promoter fragments with different lengths of –980/+120, –680/+120, –380/+120, –270/+120, and– 150/+120 were prepared from C57BL/6J brain cDNA. Each promoter fragment was produced by PCR (5 min of heat activation of the enzyme at 94°C, 40 cycles of denaturation at 95°C for 30 s, annealing at 58°C for 1 min, and extension at 72°C for 1 min) using the following primers: 5′-GAGCTCTATAGGAGGACCGGGGCAATG-3′ as –980/+120 upstream primer, 5′-GAGCTCGGCCCTTCTGCCTGACTGTCCTC-3′ as –680/+120 upstream primer, 5′-GAGCTCATTACCCTACTCCCAGGTTCC-3′ as –380/+120 upstream primer, 5′-GAGCTCCCGTTCTGCTGGCTCC-3′ as –270/+120 upstream primer, 5′-GGTACCGGATATGCCACTACGCATTCC-3′ as –150/+120 upstream primer, and 5′-CTCGAGGATGCACGCGCTGCCTGACAG-3′ as +120 downstream primer. The PCR-amplified DNA products were cloned into the pGL3-Basic Vector (Promega, Madison, WI). The 5′ end of forward primers were linked enzyme sequence of Sac1 or Kpn1, whereas the reverse primer was linked to Xho1. The 1100-, 800-, 500-, 390-, and 270-bp bands were produced by agarose gel electrophoresis. The products were digested with these linked restriction enzymes and directly ligated to the pGL3-Basic Vector. Expression vectors for dopamine receptor D1A (drd1a) and dopamine receptor D2 (drd2) were produced by ligation to pcDNA 3.1v5-His B (Thermo Fisher Scientific, MA), such as using pGL3-Basic Vector and the following primers: 5′-GGTACCGGAAGATGGCTCCTAAC-3′ as drd1a forward primer (linked 5-Kpn1), 5′-TCTAGACCAATATTCAGGTTGAATGCTG-3′ as drd1a reverse primer (linked 5 -Xba1), 5′-AAGCTTCCCAATGGATCCACTGAACC-3′ as drd2 forward primer (linked 5 -Hind3) and 5′-GATATCGACTCAGCAGTGCAGGATC-3′ as drd2 reverse primer (linked 5 -EcoR5).

### Transfection and dual luciferase assay

Dual luciferase assay was performed according to a method previously reported [18]. pGL3-Basic vector containing *Shati/Nat8L* promoter was transfected into PC12 cells using Lipofectamine 2000 (Invitrogen, Carlsbad, CA) according to the manufacturer’s recommendations. In brief, cells were incubated to confluency in 24-well plates for 24 h and exposed to a mixture of 2 μl/well of lipofectamine 2000 and 0.8 μg/well of plasmid DNA (*Shati/Nat8L* promoter-driven pGL3-Basic Vector 0.5 μg/well and CMV-Renilla luciferase 0.3 μg/well). Twenty-four hours after the transfections, the cells were incubated in a medium containing phosphate-buffered saline or METH (1 μM) for 2 h. The mediums were changed to the normal one, and then the cells were incubated for 22 h. A reporter assay was performed using the Dual-Luciferase Reporter Assay System (Promega, Madison, WI) following the instructions in the manual. The activity of CREB and NF-κB were determined same protocol as *Shati/Nat8L* promoter’s one using CRE-luc and κB-luc vectors instead of *Shati/Nat8L* vector.

### Chromatin immunoprecipitation assay

Chromatin immunoprecipitation (ChIP) was performed with mice NAc fixed by 3.7% paraformaldehyde solution for 15 min at room temperature, then resuspended in 200 μl of 1% sodium dodecyl sulfate (SDS) lysis buffers, 10 mM EDTA pH 8.0, 50 mM Tris–HCl pH 8.1 (Wako Pure Chemicals), 1 mM phenylmethylsulfonyl fluoride (Wako Pure Chemicals), and 2% protease inhibitor cocktail (Nacalai Tesque, Kyoto, Japan) and then sonicated to solubilize and shear crosslinked DNA. The suspension was centrifuged (20,000*g*, 4°C, 10 min), and then the supernatant was collected; 900 μl of ChIP dilution buffer (0.01% SDS, 1.1% TritonX-100, 1.2 mM EDTA, 16.7 mM Tris-HCl pH 8.1, 167 mM NaCl, 1 mM phenylmethylsulfonyl fluride (PMSF), 2% protease inhibitor cocktail) was then added to 100 μl of supernatant. The productions were incubated overnight at 4°C with 75l of Protein G Sepharose (GE Healthcare, Tokyo, Japan) that had been preincubated with 3 g of the appropriate antibodies {normal rabbit immunoglobulin G (IgG), CREB (48H2) rabbit mAb and NF-κB p65 (D14E12) XP Rabbit} (Cell Signaling Technology). Protein G sepharose was washed five times with low-salt buffer (0.1% SDS, 1% TritonX-100, 2 mM EDTA, 20 mM Tris-HCl pH 8.1, 150 mM NaCl), high-salt buffer (0.1% SDS, 1% TritonX-100, 2 mM EDTA, 20 mM Tris-HCl pH 8.1, 500 mM NaCl), LiCl buffer (120 mM LiCl, 0.5% NP40, 1% deoxycholate, 1 mM EDTA, 10 mM Tris-HCl pH 8.1) and twice washed with Tris-EDTA buffer (10 mM Tris-HCl pH 8.1, 1 mM EDTA). Furthermore, we extracted crosslinked DNA with 500 μl of elution buffer (10 mM dithiothreitol, 1% SDS, 0.1M NaHCO3) containing 50 mM NaCl. Bound complexes were eluted from the beads by heating at 65°C for 8 h. These elution buffers were incubated with 20 μg/ml of proteinase K (Wako, Osaka, Japan) for 2 h. Extracted DNA was then purified by phenol/chloroform/isopropanol. Purified DNA samples were normalized to 500 ng/10 μl and subjected to PCR analysis. The PCR primers were 5′-CTCGAGCCATTGTTGGAGCCAGCAGAACGG-3′ as the –270 downstream primer, and the others were the same primers as used in the luciferase assay. We analyzed PCR products by electrophoresis using 1.5% agarose gels and normalized the fluorescence of ethidium bromide to that of the control IgG.

### Western blotting

Mice were decapitated 2 h after the last METH-treatment (2 mg/kg, 6 days), and a NAc fraction was taken by brain slice. In the brain slice experiments, we also took the slices immediately after drug stimulations [artificial cerebrospinal fluid (aCSF) or METH]. NAc tissues were added to RIPA buffer (50 mM Tris-HCl pH 7.5, 152 mM NaCl, 5 mM EDTA, 1% TritonX-100, 0.5% sodium deoxy cholate, 1 mM PMSF, 2% protease inhibitor cocktail, 1% phosphatase inhibitor cocktail), sonicated on ice, and then centrifuged at 20,000*g* for 15 min at 4°C. A volume of sample buffer (312.5 mM Tris-HCl, 25% 2-mercaptoethanol, 10% SDS, 25% sucrose, 0.025% bromophenol blue) five times the supernatant volume was added. The mixture was then altered by thermal denaturation at 95°C for 5 min. The target proteins were isolated using a SDS-polyacrylamide gel electrophoresis (PAGE) method and removed from the polyacrylamide gel to a polyvinylidene fluoride membrane (Immobilon-P Trans Membrane, Merck Millipore, Darmstadt, Germany) using a semi-dry transfer method. The blots were blocked for 1 h at room temperature using 5% skim milk in Tris-buffered saline solution containing 0.1% Tween-20 (TBS-T). The membranes were incubated with polyclonal antibodies (CREB 48H2 Rabbit mAb, phospho-CREB Ser133, or 87G3 Rabbit mAb; Cell Signaling Technology) and diluted 1:1,000 in TBS-T containing 2% skim milk at 4°C for 16 h. The blots were then washed and incubated with the secondary antibody, horseradish peroxidase (HRP)-linked goat-rabbit IgG (Cell Signaling Technology). The HRP was detected using an Amersham ECL Plus Western Blotting Reagent Pack (GE Healthcare).

### Experiments using mouse brains

Brain slice experiments were performed following the protocol provided with the ChIP assay kit as described previously [19]. C57BL/6J mice were quickly decapitated, and their brains were removed and placed into ice-cold aCSF saturated with oxygen (95% O2–5% CO2 mixture, pH 7.4). Coronal mice brains were cut (400 μm) in iced aCSF and transferred to a recovery chamber containing aCSF for 50 min at room temperature. Brain slices were stimulated with METH (100 μM or 1 mM) or aCSF for 1 h after the stimulation of aCSF, PKA inhibitor KT5720 (3μM), dopamine D1 receptor antagonist SCH23390 (10 μM) or dopamine D2 receptor antagonist sulpiride (10 μM) in aCSF for 30 min, and then NAc tissues were taken for measurement of *Shati/Nat8L* mRNA. The accurate location of NAc structure was based on visual inspection of each section using a stereomicroscope and compared with the stereotaxic atlas of mouse brain [20]. NAc structures were placed on dry ice and stored at –80°C until use.

### Statistical analysis

All data were expressed as the mean ±SEM. Statistical differences between the two groups were determined using Student’s *t*-test. Statistically significant differences among values for individual groups were determined by analysis of variance, followed by the Student—Newmann–Keuls post hoc test when *F* ratios were significant (*p* < 0.05).

## Results

### *Shati/Nat8L’* mRNA was increased by METH treatment in NAc and PC12 cells

**Fig 1:**
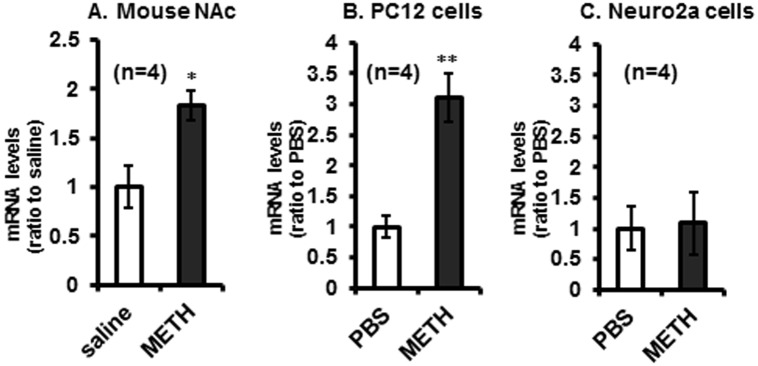
Effects of Methamphetamine (METH) on *Shati/Nat8L* mRNA in the Nucleus Accumbens (NAc) of mice and cultured cells. **(A)** *Shati/Nat8L* mRNA levels in NAc of mice repeatedly administered saline or METH (2 mg/kg/day) for 6 days. NAc samples taken 2 h after the last treatment. *n* = 4. *p < 0.05 vs saline group (Student’s *t*-test). Increasing levels of *Shati/Nat8L* mRNA induced by METH (1 μM) in (B) PC12 **(B)** and (C) Neuro2a cells. These cells were exposed to METH for 2 h. After the procedure, samples were taken for measurement of *Shati/Nat8L* mRNA **p < 0.001 vs PBS group (Student’s *t*-test). *n* = 4. Error bars represent the SEM.

Repeated administrations of METH (2 mg/kg for 6 days, s.c.) to C57BL/6J mice significantly induced *Shati/Nat8L* mRNA in NAc (*n* = 4, Fig 1A, *t*13 = 2.8793). METH (1 μM) also potentiated *Shati/Nat8L* expression 2 h after the stimulation of PC12 cells (*n* = 4, Fig 1B, *t*13 = 3.5731) but not Neuro2a cells (*n* = 4, Fig 1C). These results were consistent with those of our previous report [8, 21], which indicated that PC12 cells could upregulate *Shati/Nat8L* treated with METH. Therefore, PC12 cells were used for the luciferase assay in this study to clarify the regulatory system of *Shati/Nat8L* production.

### CREB and NF-κB -binding regions of *Shati/Nat8L* promoter were necessary for METH-induced potentiation in PC12 cells

**Fig 2:**
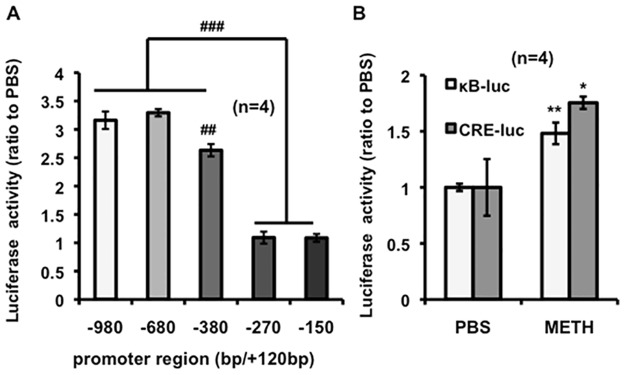
Luciferase assay using various fragments of *Shati/Nat8L* promoter regions in PC12 cells. (A) PC12 cells transfected with PGl3-Basic Vector containing five kinds of promoter region for the luciferase assay. Detection of luciferase 22 h after 2 h-methamphetamine (METH) stimulation using a Dual Luciferase Assay kit. ^##^*p* < 0.05 and ^###^*p* < 0.001 vs luciferase activities on PC12 cells comparing with −980 bp and −680 bp vector (Newman–Keuls post hoc test). *n* = 4. (**B**) METH induces activities of transcriptional factors in PC12 cells treated with METH.*p < 0.05 and **p < 0.01 vs PBS (Student’s *t*-test). *n* = 4. Error bars represent the SEM.

Because PC12 cells have dopamine transporters and tyrosine hydroxylase, the cells are useful as a model of the neuronal system [21]. Thus, the cells were used for investigation of the *Shati/Nat8L* productive system (Fig 1B). By in silico analysis on TF search, the putative binding sites of major transcriptional factors, specifically, AP-1, NF-κB, and CREB, were identified in the *Shati/Nat8L* promoter region. We designed a pGl3-Basic Vector driven by *Shati/Nat8L* promoter in which these binding sites were subsequently deleted. Luciferase activities were increased by METH on −980/+120 bp (3.16 ± 0.16-folds), −680/+120 (3.29 ± 0.66-folds) and –380/+120 (2.63 ± 0.11-folds). Promoter region of -380/+120 was decreased compared with those of -980/+120 and -680/+120. We found no activity on –270/+120 (1.09 ± 0.11-folds) and −150/+120 (1.09 ± 0.07-folds) vector 22 h after METH stimulation for 2 h (Fig 2A: −980/+120 vs −380/+120; *F*_4.15_ = 4.474, −980/+120 vs −270/+120; *F*_4.15_ = 15.88, −980/+120 vs −150/+120; *F*_4.15_ = 15.84, −680/+120 vs −380/+120; *F*_4.15_ = 4.461, −680/+120 vs −270/+120; *F*_4.15_ = 15.87, −680/+120 vs −150/+120; *F*_4.15_ = 150.83, −380/+120 vs −270/+120; *F*_4.15_ = 11.41, −380/+120 vs −150/+120; *F*_4.15_ = 11.36). The two transcriptional factors, CREB and NF-κB, were bound to the promoter region of *Shati/Nat8L* using the luciferase assay. Moreover, METH induced luciferase activities of CREB and NF-κB using CRE-luc and κB-luc vectors (CRE-luc; 1.75 ± 0.25-folds and κB-luc; 1.48 ± 0.032-folds) in PC12 cells (Fig 2B). These results suggested that METH activates CREB and NF-κB, which then induce *Shati/Nat8L* in PC12 cells.

### METH induced CREB, but not NF-κB, bind to the *Shati/Nat8L* promoter in NAc of mice

**Fig 3:**
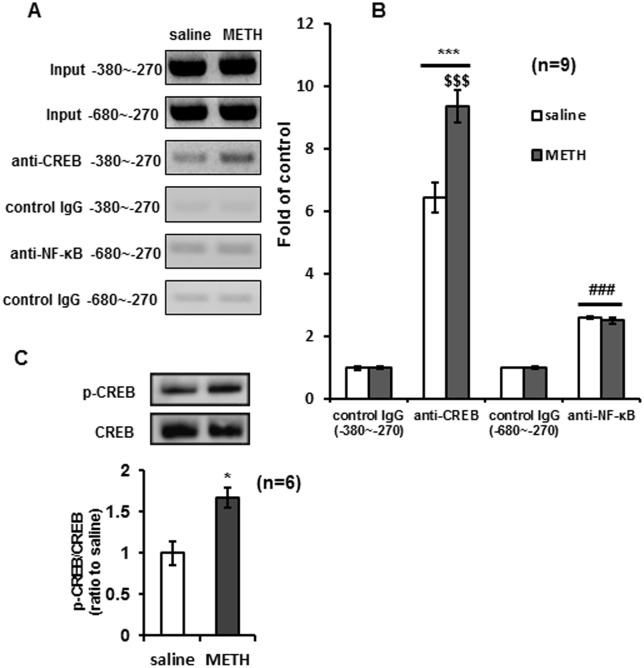
cAMP Response Element-Binding protein (CREB) binding to *Shati/Nat8L* promoter was activated by administration of Methamphetamine (METH). (**A** and **B**) ChIP (chromatin immunoprecipitation) assay was performed with antibodies for CREB and NF-κB on the nucleus accumbens (NAc) of mice repeatedly administered METH (2 mg/kg/day for 6 days. *s*.*c*.) For each group. ***p < 0.0001 vs control IgG (-380~-270), ^$$$^*p* < 0.0001 vs saline, ###*p* < 0.0001 vs control IgG (-680~-270) (Newman–Keuls post hoc test). *n* = 9 (**C**) Repeated METH potentiated the immunoreactivity of p-CREB/CREB in NAc. *p < 0.05 vs saline group (Student’s *t*-test). *n* = 6. Error bars represent the SEM.

A ChIP assay was performed on NAc tissue to identify transcriptional factors that increased Shati/Nat8L in response to METH. The assay showed that CREB and NF-κB were bound to the promoter of Shati/Nat8L. The luciferase assay was then performed (Fig 3A). However, METH induced binding of CREB but not of NF-κB to the promotor (*n* = 9, Fig 3A and 3B: saline/control IgG vs saline/anti-CREB, *F*3.32 = 18.63; METH/control IgG vs METH/anti-CREB, *F*3.32 = 28.51; saline/anti-CREB vs METH/anti-CREB, *F*3.32 = 9.961). In NAc of mice administered METH for 6 days, activation of CREB was observed (Fig 3A and 3B). Thus, repeated METH treatment of mice potentiated the phosphorylation of CREB in NAc (Fig 3C). These results suggest that METH facilitates CREB binding to the *Shati/Nat8L* promoter region from −380 bp to −270 bp.

### Increased cAMP by forskolin potentiated *Shati/Nat8L* expression in PC12 cells and brain slices

**Fig 4:**
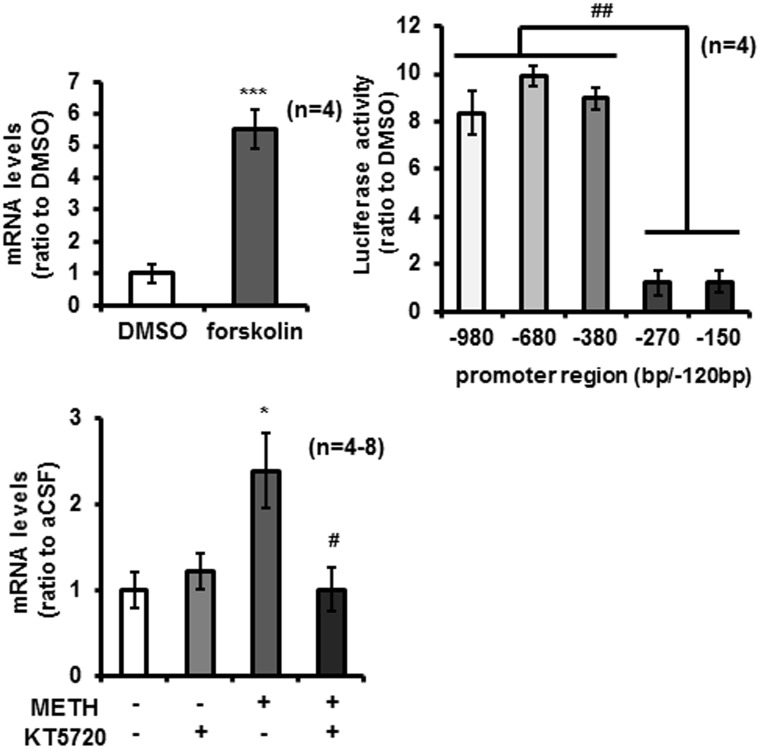
The effects of cAMP on *Shati/Nat8L* mRNA expression in PC12 cells and the nucleus accumbens slice. (**A**) PC12 cells treated with forskolin (10 μM) or DMSO as control for 2 h to perform qPCR. ***p < 0.0001 vs DMSO (Student’s *t*-test). (n = 4)(**B**) PC12 cells were transfected with PGl3-Basic Vector, including five kinds of promoter for *Shati/Nat8L* using luciferase assay. Detection of luciferase performed 22 h after 2-h forskolin stimulation (10 μM) using a Dual Luciferase Assay kit. ^##^*p* < 0.001 vs –980/+120, −680/+120, and −380/+120 vector (Newman–Keuls post hoc test). (n = 4) (**C**) Brain slices treated with a PKA inhibitor, KT5720 (3 μM), for 30 min before methamphetamine (METH) treatment (1 mM) to perform qPCR. *p < 0.05 vs aCSF, ^#^*p* < 0.05 vs METH (Newman–Keuls post hoc test). (n = 4–8) Error bars represent the SEM.

The ChIP assay results showed that CREB was necessary for potentiation of *Shati/Nat8L* expression. To investigate the upstream of CREB in the Shati/Nat8L site, we focused on cAMP, which activates PKA following CREB activity. Stimulation of forskolin (10 μM), an inducer for cAMP signaling, increased *Shati/Nat8L* mRNA in PC12 cells (Fig 4A). Moreover, the luciferase assay results supported the possibility that CREB induced *Shati/Nat8l* expression in PC12 cells (Fig 4B). Furthermore, stimulation of KT5720 (3 μM) were significantly inhibited the expression of *Shati/Nat8L* mRNA expression induced by METH in mice brain slices (Fig 4C: aCSF vs METH, *F*3.20 = 4.233; METH vs METH+KT5720, *F*3.20 = 4.553). These results suggested that CREB activity for *Shati/Nat8L* expression is caused by cAMP increases.

### *Shati/Nat8L mRNA* expression was increased by METH via dopamine D1 receptor signaling to CREB

**Fig 5:**
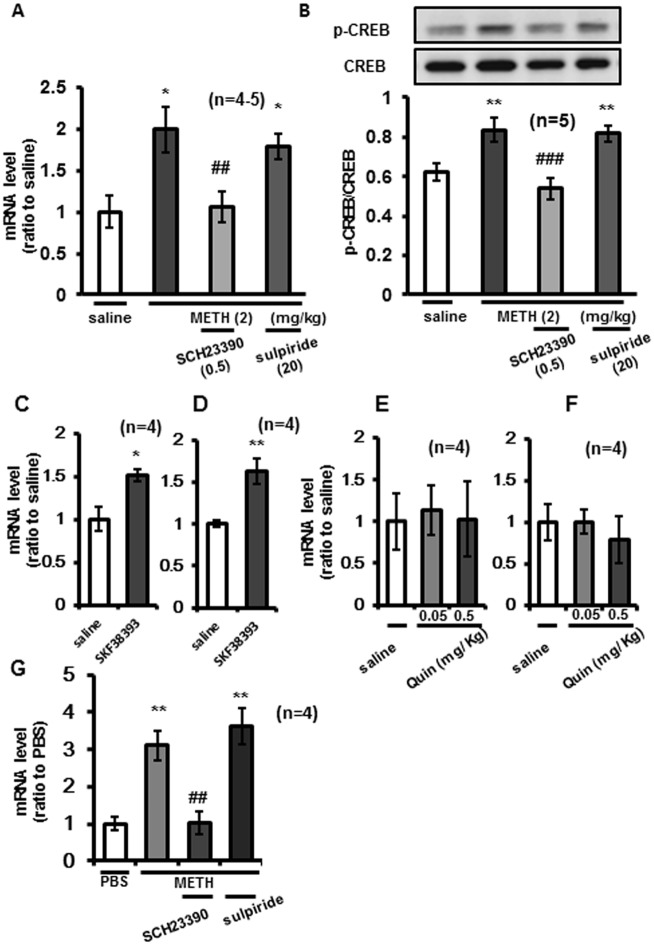
Dopamine D1 receptor potentiated expression of *Shati/Nat8L* mRNA by Methamphetamine (METH) in the Nucleus Accumbens (NAc). (**A**) The effects of pharmacological inhibition by dopamine D1 (SCH23390, 0.5 mg/kg) or D2 (sulpiride, 20 mg/kg) receptor antagonist in mice NAc were analyzed by qPCR. Dopamine D1 receptor antagonist inhibits METH-induced increases in *Shati/Nat8L* mRNA in NAc. *p < 0.05 vs saline, ^##^*p* < 0.01 vs METH effects (Newman–Keuls post hoc test). n = 4–5. (**B**) Phosphorylation of cAMP response element-binding protein (CREB) in mice NAc were assayed by Western blotting. **p < 0.01 vs saline, ^#^*p* < 0.005 vs METH effects (Newman–Keuls post hoc test) n = 5. The effects of Shati/Nat8L mRNA in mice NAc by (**C**) single and (**D**) repeated administrations of dopamine D1 receptor agonist, SKF38393 (0.5 mg/kg s.c.) were analyzed by real time RT-PCR. *p < 0.05 and **p < 0.01 vs saline in each condition (Student’s -*t*-test). n = 4. The influences of (**E**) single (n = 4) and (**F**) repeated administration of dopamine D2 agonist quinpirole hydrochloride (Quin) (0.05 and 0.5 mg/kg s.c.) assayed by qPCR. n = 5. (G) Dopamine D1 or D2 receptor antagonist (D1: SCH23390 10μM, D2: sulpiride 10μM) were pretreated before METH (1mM) stimulation in PC12 cells, followed by qPCR for Shati/Nat8L mRNA expression was determined. **p < 0.01 vs PBS, ^##^*p* < 0.05 vs METH effects (Newman–Keuls post hoc test). n = 4. Error bars represent the SEM.

To examine the upstream of METH-induced CREB activity, experiments involving pharmacological inhibition of METH activity were performed. Because it is well known that METH increases dopamine levels [3, 4], we focused on dopamine receptor signals. Pretreatment with SCH23390 (0.5 mg/kg *s*.*c*.), but not sulpiride (20 mg/kg s.c.), inhibited METH-induced increases in *Shati/Nat8L* mRNA in NAc (Fig 5A: saline vs METH, *F*3.15 = 4.483; saline vs sulpiride + METH, *F*3.15 = 3.074; METH vs SCH23390+METH, *F*3.15 = 3.706). In addition, dopamine D1 receptor antagonist inhibited METH-induced CREB activity in NAc (Fig 5B: saline vs METH; *F*3.16 = 5.021, saline vs sulpiride + METH *F*3.16 = 4.386, METH vs SCH23390+METH *F*3.16 = 6.852). To determine the degree that dopamine D1 or D2 receptors contributed to the METH-related activity, we injected D1 receptor agonist or D2 receptor agonist into mice (*s*.*c*.). Thus, single- or repeated dopamine D1 receptor agonist SKF38393 (0.5 mg/kg, s.c.) administration potentiated *Shati/Nat8L* mRNA expression in NAc of mice (Fig 5C and 5D). However, dopamine D2 agonist quinpirole hydrochloride had no effects on *Shati/Nat8L* mRNA expression in NAc (Fig 5E and 5F). Alternatively in PC12 cells SCH23390, but not sulpiride, inhibited the increase in *Shati/Nat8L* mRNA (Fig 5G: PBS vs METH; *F*3.12 = 6.291, PBS vs sulpiride + METH; *F*3.12 = 5.734, METH vs SCH23390+METH; *F*3.12 = 5.554). These results supported a putative mechanism in which *Shati/Nat8L* expression is regulated by activation of dopamine D1 receptor signaling.

## Discussion

*Shati/Nat8L* has been shown to be increased in NAc of mice after repeated treatment of METH [8]. Important neuronal roles of *Shati/Nat8L*, in addition to the function of lipid turnover in brown adipocytes, were subsequently demonstrated [11, 12, 14, 22, 23]. Recently, it was reported that NAA produced from *Shati/Nat8L* was associated with Canavan disease [24]. Although these reports have shown that *Shati/Nat8L* has important physiological functions in the central nervous system and peripheral tissues, the regulatory mechanism of Shati/Nat8L expression has remained unknown. In the present study, we attempted to elucidate the mechanism of Shati/Nat8L production at the molecular level.

First, we reconfirmed the activation of *Shati/Nat8L* mRNA expression in NAc of mice repeatedly treated with METH (Fig 1A). This finding of inductive activity agreed with our previously reported findings [8]. We also demonstrated that the increase in *Shati/Nat8L* mRNA by METH was regulated by CREB activity via dopamine D1 receptor signaling in NAc of mice (Figs 3–5). Because PC12 cells have DAT, Tyrosine hydroxylase, and dopamine receptors, the cells mimicked NAc of mice [7]. Moreover, forskolin, an inducer of cAMP in cells, also mimicked METH-induced luciferase activity and *Shati/Nat8L* mRNA induction (Fig 4). These results indicated that *Shati/Nat8L* expression depended on the CREB and cAMP pathway. *Shati/Nat8L* mRNA was not increased by treatment with METH at Neuro2a (Fig 1B) because Neuro2a has not been considered to have tyrosine hydroxylase and D1R [25, 26]. Furthermore, overexpression of the drd1a gene, but not the drd2 gene, in Neuro2a induced *Shati/Nat8L* mRNA (S1 Fig). These results also support our conclusion that Shati/Nat8L production is dependent on the dopamine D1 receptor pathway.

We have previously reported that *Shati/Nat8L* overexpression in NAc controlled the dopaminergic system via activation of mGluR3 by elevated NAAG following NAA production [11]. We had thought that overexpression of Shati/Nat8L in Nac interneurons indirectly affected sensitization to METH and dopamine release to NAc. Nevertheless, a previous report indicated that METH-induced *Shati/Nat8L* potentiation occurred at D1-MSN, which directly released GABA to the GABA interneurons of VTA [7]. Thus, D1R-signal-potentiated NAAG appeared to suppress GABA release from D1-MSN to GABA interneurons in VTA via activation of mGluR3 at D1-MSN.

Previously, we reported that Shati/Nat8l knockout caused D1R over localization on cell surfaces in NAc of mice and that the mice exhibited stronger responses to SKF38393 and METH than did WT mice [14]. Consistent with the present and previous results, overexpression of dominant negative CREB in D1R-expressed cells has been shown to increase the response to cocaine [27]. These results support our finding that *Shati/Nat8L* expression had a suppressive role in drug addiction downstream of dopamine D1 receptor signaling. Furthermore, these reports also indicated that *Shati/Nat8L* expression by CREB on D1R signaling was regulated via a cycle involving D1R signaling to CREB activity, CREB to *Shati/Nat8L* expression, and *Shati/Nat8L* to D1R localization.

In the present study, we also showed that *Shati/Nat8L* expression was regulated by NF-κB. Exogenous TNF-alpha in NAc has been shown to attenuate METH-induced addiction [28]. Previously, we also showed that *Shati/Nat8L* overexpression in PC12 cells increased TNF-alpha mRNA and also that *Shati/Nat8L* KO mice had a lower level of LITAF upstream of TNF-alpha in the brain [10]. The association of NF-κB with *Shati/Nat8L* promotor using luciferase assay could be a secondary effect (Fig 2A and 2B). The finding indicates that the promoter activity of NF-κB were regulated by TNF-alpha due to *Shati/Nat8l* upregulation induced by METH stimulation. These results indicated that the *Shati/Nat8L* gene may be associated with the cytokine family.

In conclusion, we demonstrated the importance of the *Shati/Nat8L* promoter region and of CREB to *Shati/Nat8L* expression, and that expression of *Shati/Nat8L* mRNA was regulated especially by activation of CREB. These results suggest that inactivation of this mechanism may be a risk factor of addiction.

## Supporting information

S1 Fig The relationship of drd1a gene to *Shati/Nat8L* expression. The Figure indicates that the relationship between Shati/Nat8L expression and METH effect on brain slice experiments. **p<0.01 *vs*. aCSF. (Newman–Keuls post hoc test). (B) Dopamine D1 or D2 receptor antagonist (D1: SCH23390 10uM, D2: sulpiride 10uM) was pretreated before METH (1mM) stimulation for qPCR of Shati/Nat8L mRNA. **p<0.01 and ***p<0.001 *vs*., aCSF## *p*<0.01 *vs*.METH. (Newman–Keuls post hoc test). (C) PC12 cells transfected with drd1a or drd2 were taken for qPCR 48 after the transfection * *p*<0.05 vs pcDNA (Newman–Keuls post hoc test). Error bars represent the S.E. M
(TIF)
