# Supplementary figures and images for: Methamphetamine induces Shati/Nat8L expression in the mouse nucleus accumbens via CREB- and dopamine D1 receptor-dependent mechanism
Source: PLoS One. 2017 Mar 20;12(3):e0174196. doi: 10.1371/journal.pone.0174196 (PMC5358781; doi:10.1371/journal.pone.0174196)

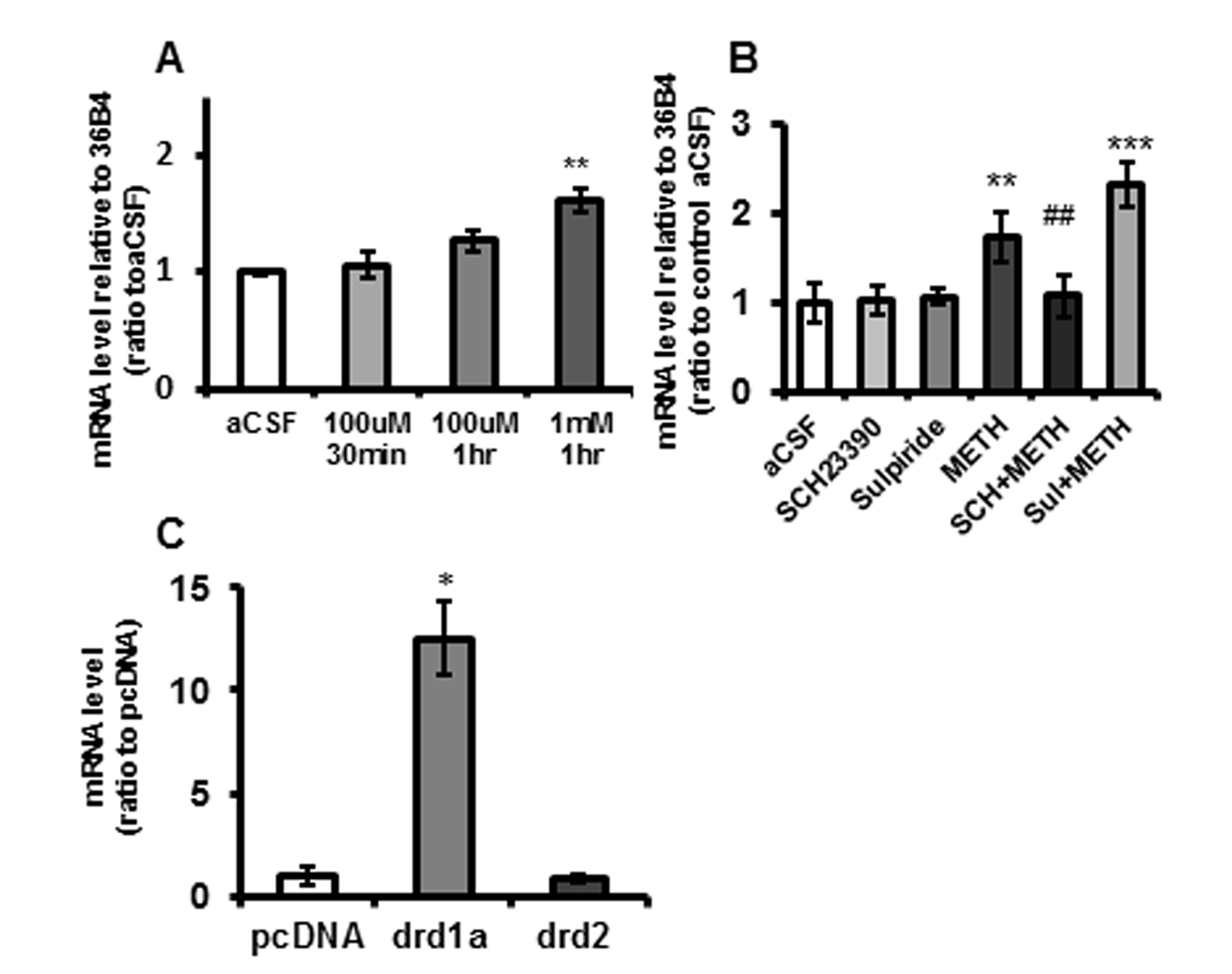

Supplement: S1 Fig — The Figure indicates that the relationship between Shati/Nat8L expression and METH effect on brain slice experiments. **p<0.01 vs. aCSF. (Newman–Keuls post hoc test). (B) Dopamine D1 or D2 receptor antagonist (D1: SCH23390 10uM, D2: sulpiride 10uM) was pretreated before METH (1mM) stimulation for qPCR of Shati/Nat8L mRNA. **p<0.01 and ***p<0.001 vs., aCSF## p<0.01 vs.METH. (Newman–Keuls post hoc test). (C) PC12 cells transfected with drd1a or drd2 were taken for qPCR 48 after the transfection * p<0.05 vs pcDNA (Newman–Keuls post hoc test). Error bars represent the S.E. M (TIF) [file pone.0174196.s001.tif]
